# Can Syphilis Be Transmitted by Means of the Spermatic Fluid?

**Published:** 1878-07

**Authors:** 


Can Syphilis be Transmitted by means of the Spermatic Fluid.—A contribution to this much debated topic is to be found in the *Annales de Dermatologie et de Syphiligraphie*, tome 8, No. 6. It is in the form of an original article by Dr. H. Mireur, known as the author of an admirable thesis entitled "*Essai sur l' Heredite de la Syphilis.*" In this article Mireur, after showing the inadequacy of the several observations which have been brought forward to prove the inoculability of the spermatic fluid of a syphilitic person, adduces several cases coming under his own notice, in which inoculation was attempted without success. A patient in full evolution of secondary syphilis, having roseola papulosa, mucus patches, etc., provided fresh spermatic fluid, which was immediately inoculated upon four persons absolutely free from syphilitic antecedents. Upon two the spermatic was introduced into the arm by charged needles. Upon a third a blister was produced upon the leg the size of a ten-cent piece, and a charpie soaked with the spermatic fluid placed on the raw surface. On the arm of the fourth person an abrasion was made over the insertion of the deltoid, and several transverse incisions were made at this spot. The matter to be inoculated was placed upon this abraided surface as in the previous cases. With the exception of a slight local inflammation, no result whatever ensued; no symptoms of syphilis of any kind were noticed. Mireur points out the frequency of the contagion by the blood and the secretion of mucus patches, comparing the statistics of this variety of infection with those adduced in that under consideration, and analyzes the assertions of the writers who maintain the infectiousness of the spermatic fluid. These will not bear close examination at all, and we are inclined to think that Dr. Mireur has so far decidedly the advantage in the strength of the proofs he brings forward. *Philadelphia Medical Times.*

